# β-Sheet Breaker Peptide-HPYD for the Treatment of Alzheimer's Disease: Primary Studies on Behavioral Test and Transcriptional Profiling

**DOI:** 10.3389/fphar.2017.00969

**Published:** 2018-01-08

**Authors:** Weiying Liu, Fengxian Sun, Moxin Wan, Fang Jiang, Xiangyu Bo, Laixiang Lin, Hua Tang, Shumei Xu

**Affiliations:** ^1^Department of Pathogen Biology, Tianjin Life Science Research Center, School of Basic Medical Sciences, Tianjin Medical University, Tianjin, China; ^2^Department of Physiology and Pathophysiology, School of Basic Medical Sciences, Tianjin Medical University, Tianjin, China; ^3^Department of Pathology, Institute of Hematology and Blood Diseases Hospital, Chinese Academy of Medical Science and Peking Union Medical College, Tianjin, China; ^4^Key Laboratory of Hormone and Development (Ministry of Health), 2011 Collaborative Innovation Center of Tianjin for Medical Epigenetics, Metabolic Diseases Hospital and Tianjin Institute of Endocrinology, Tianjin Medical University, Tianjin, China

**Keywords:** Alzheimer's disease, HPYD, β-amyloid peptides, APP, β-sheet breaker peptide

## Abstract

**Background:** Alzheimer's disease (AD), is a progressive neurodegenerative disease that is characterized by cognitive loss. Most researchers believe that aggregation and accumulation of β-amyloid peptides (Aβ) in brain cells are the central pathological hallmark of this disease.

**Methods:** Based on the amyloid hypothesis, a 10 amino acids β-sheet breaker peptide HPYD (His-Lys-Gln-Leu-Pro-Phe-Tyr-Glu-Glu-Asp) was designed according to the structure and sequence of the previous designed peptide H102. Accelerated stability test, thioflavine T (ThT) fluorescence spectral analysis and transmission electron microscopy (TEM) imaging were performed to detect the stability and inhibitory effects on the aggregation of Aβ_1−42_ by H102 and HPYD. FITC-labeled HPYD was first tested to determine whether it could be transferred along the olfactory pathway to the brain after nasal administration to mice. Subsequently, the Morris Water Maze (MWM) test for behavioral analysis was used to investigate the learning and memory ability of APP/PS1 transgenic mice by HPYD. Immunohistochemistry and western blot analysis was performed to determine the role of HPYD on Aβ and APP protein levels. In addition, microarray analysis was used to evaluate the effect of HPYD on gene expression in AD mouse models.

**Results:** Our *in vitro* results demonstrated that HPYD had enhanced stability and inhibitory effects on Aβ_1−42_ aggregation compared to H102. HPYD could be delivered into the brain through nasal administration and improved the learning and memory ability in APP/PS1 transgenic mouse models by reducing Aβ and APP protein levels. In addition, microarray analyses suggested that several genes related to the inflammatory pathway, AD and gluco-lipid metabolism were dysregulated and could be restored to almost normal levels after HPYD administration to mice.

**Conclusions:** Our results demonstrated that HPYD could be a potential therapeutic drug candidate for the treatment of AD.

## Introduction

Alzheimer's disease (AD) is a complex, severe neurodegenerative disorder of the central nervous system, which manifest as progressive cognitive decline and memory impairment in the elderly and presenium individuals. More than 44 million people are currently living with AD and the global health care cost in 2010 was about US$818 billion (Weiner et al., 2013). The incidence of AD has been increasing every year, and by 2050 about 131.5 million people will suffer from dementia if there are no effective therapies (Cummings et al., 2016). Although AD has led to a major social and health care problem, there is still no absolute effective treatment for the disease. Hence, it is critical to develop novel drugs and effective therapies for AD.

The pathological characteristics of AD mainly include massive senile plaque deposits, neurofibrillary tangles as well as selective loss of neurons and synapses in specific brain regions, such as the cerebral cortex and hippocampus (Mattson, 2004; Shen and Kelleher III, 2007). Senile plaques contain extracellular deposits of β-amyloid peptide in its fibrillar form and neurofibrillary tangles, which are composed of hyper phosphorylated tau protein. There are many hypothesis regarding the pathogenesis of AD, such as the cholinergic hypothesis (Francis et al., 1999), amyloid hypothesis (Hardy and Allsop, 1991; Mudher and Lovestone, 2002), tau hypothesis (Goedert et al., 1991; Mudher and Lovestone, 2002) and several other hypotheses (Reisberg et al., 1999; Deane and Zlokovic, 2007). The amyloid hypothesis has become the most accepted mechanistic hypothesis for AD pathogenesis, although some amendments have been made.

β-amyloid peptides (Aβ) are 39–42 amino acid peptide residues that are derived from putative intramembranous processing of amyloid precursor protein (APP) by γ-secretase/PS1 aspartyl protease (Selkoe, 1999; Hardy and Selkoe, 2002). Only a small amount of Aβ is generated by the cleavage of APP by β- or γ-secretase in healthy individuals, while a large amount of APP is metabolized by α- and β-secretase without Aβ generation (Haass et al., 1992; Zhang and Xu, 2007). However, genetic mutations in β- or γ-secretase in AD patients results in increased APP cleavage activity, which subsequently generates large amounts Aβ. Aβ has been shown to aggregate and accumulate abnormally in the brain of AD patients, and extracellular amyloid plaques of Aβ peptides aggregation can trigger a cascade of pathologic events leading to nerve fiber entanglement and neuronal apoptosis (Hardy and Selkoe, 2002; Karran et al., 2011).

Recently, it has been demonstrated that smaller and more soluble aggregates, including a variety of compounds of soluble oligomers, ADDLs (amyloid β-derived diffusible ligands) or protofibrils to be the predominant toxic forms of Aβ (Walsh and Selkoe, 2007; Allsop and Mayes, 2014; Karran and De Strooper, 2016; Selkoe and Hardy, 2016). Compared to fibrillar plaques, Aβ oligomers (AβOs) are considered to be the main mediators of cytotoxicity in AD (Ashe and Aguzzi, 2013). The oligomeric Aβ can initiate the phosphorylation of Src kinase Fyn, asparagine endopeptidase (AEP) and GSK3-β, which then can subsequently induce the hyperphosphorylation of tau protein (Martin et al., 2013; Zhang et al., 2014). AβOs and ADDLs bind to synaptic contacts and cellular membranes more rapidly and with higher affinity than fibrillar Aβ (Aβf). This binding compromises the integrity of intracellular membranes to induce an elevation of intracellular Ca^2+^, which then results in rapid and massive neuronal cell death (Demuro et al., 2005; Deshpande et al., 2006). In addition, Aβ also could upregulate inflammatory cytokines and increase the nitric oxide release to cause neuroinflammation (Hu et al., 1998). Several studies have demonstrated that Aβ can induce the expression of several inflammatory factors, including CASP1, CASP4, PLA2G4A, and PTPRC (Lee et al., 2011; Zhu et al., 2011; Mehta et al., 2012; Kajiwara et al., 2016). It has been reported that soluble Aβ_1−42_ protofibrils could stimulate microglial production of tumornecrosis factor α (TNFα) to stimulate inflammatory responses (Paranjape et al., 2013). Taking all these observations and studies into consideration, the amyloid hypothesis is recognized as the most prominent theory to explain the pathogenesis of AD.

It is thought that Aβ aggregations are generated due to Aβ clearance deficiencies of γ-secretase (Jarrett et al., 1993; Golde et al., 2000; McGowan et al., 2005). Hence, many secretase inhibitors were developed as the initial small-molecular therapies for AD (De Strooper et al., 2010). Semagacestat, a γ-secretase inhibitor, reached Phase 3 clinical trials, but was halted due to adverse events of worsening cognition and activity, as well as increasing the incidence of skin cancer (Karran and De Strooper, 2016). Many other strategies were also considered for AD, for example, inhibiting Aβ aggregation. Tramiprosate was found to inhibit Aβ aggregation by maintaining Aβ in a non-fibrillar form, thus inhibiting amyloid deposition (Gervais et al., 2007). Tramiprosate could significantly reduce brain amyloid plaque load (~30%) and the cerebral levels of soluble and insoluble Aβ_40_ and Aβ_42_ (~20–30%) in TgCRND8 mice (Gervais et al., 2007). However, no significant therapeutic effects were observed as primary outcomes in the Alzheimer Disease Assessment Scale-cognitive subscale (ADAS-cog) and Clinical Dementia Rating-Sum of Boxes (CDR-Sum of Boxes) in phase III trials (Aisen et al., 2011). Nonetheless, ALZ-801, a novel prodrug of tramiprosate, has shown excellent oral safety and tolerability, and its PK characteristics were significantly improved compared to oral tramiprosate in phase I studies (Hey et al., 2017). Additionally, immunotherapies were considered for AD therapy. Bapineuzumab and solanezumab represent two humanized monoclonal antibodies that increases the clearance of Aβ by specifically targeting amino acids 1–5 and 16–24 of Aβ peptide, respectively. However, Phase 3 clinical trials of bapineuzumab were halted after the completion of two trials because it did not improve clinical outcomes in patients with AD (Salloway et al., 2014). Similarly, solanezumab also failed to meet the primary outcome in Phase 3 clinical trials (Siemers et al., 2016). Although many amyloidocentric drugs have failed after Phase 3 clinical trials, it does not diminish the pathogenic theory of this disease.

Aβ consists of a hydrophobic carboxyl terminus and a hydrophilic amino terminus. The hydrophobic carboxyl terminus of Aβ mainly consists of β-sheets while the hydrophilic amino terminus mainly consists of α-helix and β-turns (Chou and Fasman, 1977). Aggregation of monomeric Aβ into oligomers are formed through the internalization of the hydrophobic carboxyl terminus and exposing the hydrophilic amino terminus (Hilbich et al., 1992). The carboxy terminus of Aβ is critical for amyloid formation.

β-sheet breaker peptides (also referred to as peptidic inhibitors) are a class of compounds that are highly potent in ameliorating Aβ_1−42_- or α-synuclein-inflicted cell toxicity (Watanabe et al., 2002; El-Agnaf et al., 2004). β-sheet breaker peptides are homologous to regions of the β-sheet hydrophobic carboxyl segments and highly effective in inhibiting Aβ amyloidogenesis (Jarrett et al., 1993). Based on the amino acid residues 17–21 of Aβ_1−42_, we previously designed a β-sheet breaker peptide H102 (His-Lys-Gln-Leu-Pro-Phe-Phe-Glu-Glu-Asp) that can reduce amyloid load and cerebral damage and improve the learning and memory ability of AD animal models (He et al., 2008; Lin et al., 2014). However, H102 was not very stable. Hence, we designed an alternative β-sheet breaker peptide HPYD (His-Lys-Gln-Leu-Pro-Phe-Tyr-Glu-Glu-Asp) by substituting Phe with Tyr. The stability of HPYD and the ability to inhibit Aβ aggregation were studied *in vitro*. HPYD displayed excellent stability and the ability to inhibit Aβ aggregation compared to H102. However, HPYD efficacy for the treatment of AD *in vivo* remained to be elucidated.

In this study, we first compared the stability of H102 and HPYD using the accelerated stability test, and then performed inhibitory studies on the aggregation of Aβ_1−42_. We also investigated the ability of HPYD to transverse into the brain through the olfactory pathway after nasal administration of fluorescein isothiocyanate (FITC)-labeled HPYD. The effect of HPYD on APP/PS1 transgenic mice behavior and the APP and Aβ expression in the brain were also investigated. Furthermore, we profiled the gene expression in normal mice (control group), APP/PS1 transgenic mice (model mice) and APP/PS1 transgenic mice treated with HPYD (HPYD group) using gene microarrays. Gene ontology (GO) analysis and Kyoto Encyclopedia of Genes and Genomes (KEGG) pathway analysis were performed to annotate their functions. Our findings not only has important implications for the potential treatment of AD using HPYD, but also provide insights to the mechanism of Aβ toxicity in AD patients and for the development of new therapeutic strategies for AD.

## Materials and methods

### Animals

Nude mice (weight: 26 to 28 g; age: 2 months), APP/PS1 mice (weight: 25.3 to 28.1 g; age: 8 months) and C57/6J mice (weight: 25.5 to 28.6 g) were purchased from the institute of laboratory animal sciences, CAMS & PUMC (Chinese Academy of Medical Sciences and Peking Union Medical College). APP/PS1 mice have been previously demonstrated to form amyloid plaques, which have been approved by institute of laboratory animal sciences, CAMS and PUMC. All animal experiments were performed in accordance with the China Physiological Society “Guiding Principles in the Care and Use of Animals” approved by Tianjin Medical University Animal Care and Use Committee (NO. 20130021).

### Compounds

H102, FITC-labeled HPYD and HPYD were synthesized using the Fmoc solid-phase synthesis method and purified by HPLC (Gill Biotechnology Company, Shanghai, China). The compounds were greater than 95% pure as measured by HPLC-MS. HPYD, a polypeptide comprising the amino acid (AA) sequence of His-Lys-Gln-Leu-Pro-Phe-Tyr-Glu-Glu-Asp, was dissolved in normal saline.

### Accelerated stability test

0.5 mg of HPYD or H102 was dissolved in 1 mL of 0.2 mol/L phosphate buffer containing 100 mg/L of trypsin. The trypsin solution and polypeptide were mixed at a ratio of 1:4, and the solution was placed in a temperature controlled water bath shaker at 37°C for 0, 40, 100, 160, 220, 280, 340, and 400 min. The samples were then heated to 80°C for 10 min, and the stability of HPYD and H102 was detected at the different time points. High performance liquid chromatography (HPLC) analysis of HPYD and H102 was performed using a Phenomerex C18 column (250 mm × 4.6 μm, 5 μm; Sigma, Inc. U.S.A.). The mobile phase consisted of solvent A, 0.1% TFA in acetonitrile and solvent B, 0.1% TFA in water at a ratio of 22.5:77.5 (v/v). The sample injection volume was 20 μL. The flow rate was 1 mL/min, and the detection wavelength was 220 nm.

### Thioflavine T (ThT) fluorescence spectral analysis

Aβ_1−42_ freeze-dried powder was dissolved in 50 mmol/L sodium phosphate buffer solution (pH = 7.4) to a concentration of 22.15 μmol/L, and H102 and HPYD were dissolved in the same PBS solution to a concentration of 88.60 μmol/L. Aβ_1−42_ solution was then mixed with H102 and HPYD respectively in equal volumes. The Aβ fibrils were grown at 37°C for 24 hrs. 10 μL solution from each group was then added to 990 μL of 3.0 μmol/L ThT solution and fluorescent intensity was measured using a VARIAN PTC-Au00-01058 fluorescence spectrophotometer (VARIAN, USA) with an excitation wavelength of 453 nm and emission of 478–486 nm.

### Transmission electron microscopy (TEM)

The Aβ_1−42_ samples (11.07 μmol/L 20 μL) were incubated in 37°C for 5 days, and the mixture of Aβ_1−42_ (22.15 μmol/L 10 μL) with HPYD (88.61 μmol/L 10 μL) or H102 (88.61 μmol/L 10 μL) were incubated under the same conditions for 5 days. Aliquots (5 μL) of each sample was spotted onto thin carbon substrates supported by carbon film on a 300 mesh copper grid for 15 min and blotted dried. The TEM grids were negatively stained with 2% uranyl acetate for 2 min and air dried. The samples were subsequently imaged using a Hitachi H-600 transmission electron microscope.

### Fluorescence imaging system analysis

Nude mice were anesthetized using 20% urethane (5 mL/kg), and then placed on the VFIS observation platform. Nude mice were then irradiated at 490 nm excitation without treatment, and then irradiated for 5, 15, and 30 min after nasal administration with FITC-HPYD (5.535 mg/kg). Thirty minutes after nasal administration with FITC-HPYD, the mice were euthanized and the hippocampus, cortex, olfactory bulb, heart, lung, liver, spleen and kidneys were removed. These organs were then irradiated at 490 nm excitation.

### HPYD treatment

The APP/PS1 transgenic mice were randomized into the model and HPYD treatment group (*n* = 12 for each group). C57BL/6J mice with the same genetic background and age served as the normal controls. Treatment group received intranasal administration of HPYD saline solution (33 mg/mL, 5 μL/d), while the normal control group and model group were given with the same volume of saline for 30 days.

### Behavioral test

The behavioral test was performed after 30 days of intranasal administration using the Morris Water Maze (MWM) according to our previous report (Lin et al., 2014). Briefly, the MWM test was conducted in a circular pool with a diameter of 80 and 32 cm deep. The pool was filled with 25 ± 1°C water to a depth of 1 cm higher than the escape platform. The water was made opaque white to hide the escape platform. The orientation and navigation experiments were conducted for 5 days to record the time intervals when the animals climbed onto the platform. If animal failed to find the hidden platform within 90 s, the mouse was placed back onto the platform for 20 s, and the escape latency was recorded as 90 s. The platform was placed in the center of the third quadrant and remained in the same position throughout the orientation and navigation experiment. On the 6th day, the platform was removed, and each mouse was allowed to swim freely for 90 s to record the frequency of passing the hidden platform and the original angle.

### Immunohistochemistry

At the end of the MWM test, the mice were euthanized and their brains were removed and rapidly placed on ice. The brain tissues were then fixed in 4% paraformaldehyde solution and waxed. Tissue sections were then dewaxed, and antigen retrieval was performed using boiling citrate buffer solution. This was followed by incubating the sections in 3% H_2_O_2_ solution at room temperature for 10 min to block endogenous peroxidase activity. The sections were then blocked by incubating with normal goat serum at room temperature for 15 min, and subsequently diluted antibodies (Aβ: 1:100; APP: 1:100) were added and incubated overnight at 4°C. Biotin-labeled secondary antibody was added and incubated for 15 min at room temperature, followed by incubation with strept avidin-biotin complex (SABC) and DAB chromogenic reagent. Finally, the sections were counterstained with hematoxylin and observed under a microscope.

### Western blotting

Western blot analysis was performed according to the detail procedures described previously. Primary Aβ antibody (1:200) was purchased from Abcam (USA) and anti-APP antibody (1:200) was purchased from Wuhan BOSTER Bio Company (Wuhan, China). The secondary goat anti-rabbit antibody was purchased from Sigma-Aldrich (St Louis, MO, USA).

### Microarray analysis

Microarray hybridization was carried out by Shanghai GMINIX Biotech Limited Company (China) using the GeneChip® Mouse Gene 1.0 ST Array (Affymetrix, USA Scientific) with 770,317 probes. Briefly, total RNA was extracted from frozen brain tissues using the RNeasy mini kit (Qiagen, Valencia, CA) and used for cDNA synthesis using the cDNA synthesis kit (Affymetrix, Inc., USA). cDNA was labeled using the Gene Chip2 WT Terminal Labeling Kit (Affymetrix, Inc., USA), and then the labeled cDNA was hybridized to the mouse Gene chip at 45°C for 16 h. The Gene chip was then washed with wash solution A, wash solution B and deionized water, and stained using Cocktail 1 and Cocktail 2. The Gene Chip 2 Scanner 300 7G (Affymetrix Inc.) and the AGCC Scan Control software was used for date analysis. The gene ontology (GO) enrichment analysis was performed using the GOEAST software toolkit (*P* ≤ 0.05), and signaling pathway analysis was performed using the KEGG data software. Software Matlab 7.1 and java, was used to build and analyze the dynamic Gene networks, and network maps of the differentially expressed genes were constructed from the different states.

### Accession numbers

The Gene Expression Omnibus accession number for normal mice, model mice and HPYD mouse expression profiles is GSE104249.

### Real-time quantitative PCR (RT-qPCR)

RT-qPCR analysis was performed using an ABI 7500 thermocycler (Applied Biosystems) with UltraSYBR Mixture purchased from Beijing ComWin Biotech Co., Ltd. (Beijing, China). Gene transcript normalization was performed using housekeeping gene β-actin. All primers used for RT and qPCR are listed in Table 1.

**Table 1:** Primers used in this study.

**Name**	**Sequence (5′-3′)**
PLA2G4A-qF	AGAAGGACGTGCCGGAAAGGTGCAT
PLA2G4A-qR	CCGCTGCGTCGAGCTCGTCATCGAA
CLEC4D-qF	TACCACACGAGAGTAACGTGCATCC
CLEC4D-qR	CTCTCATGCCAGGTCTGGTTGTCAT
APP-qF	ACCGGGCCATGCCGCGCAATGATCT
APP-qR	GCCACACACCGCCATGCAGTACTCT
PTPRC-qF	AACTGAGCACAACAGAGAATGCCCT
PTPRC-qR	AGCGTGGATAACACACCTGGATGAT
CDK1-qF	GACTTGAAAGCGAGGAAGAAGGAGT
CDK1-qR	TCAAAGATGAGATACAGCCTGGAGT
CASP1-qF	TCTGGGGTATACCGTGAAAGTGAAA
CASP1-qR	GATACCATGAGACATGAATACAAGG
SCN9A-qF	TGCCCTCATTGAACAACGCATTTCT
SCN9A-qR	TCTCCGTAGATGAAGGGTAGCTGTT
FCGR4-qF	CCCCAAGTGGGTCAGGGTGCTTGAG
FCGR4-qR	TGGGTCACTGATCGTGGAGAGGGCT
CLEC5A-qF	GACGAAAGTACCATGCCTACAAGGA
CLEC5A-qR	TGGAGTGTTGACAATTGCCAGTGTG
DDX3X-qF	AAGGGCGTTATATCCCACCTCATTT
DDX3X-qR	AAGAAGCTAGACTTCCCTCTTGAAT
CASP4-qF	ACGCAGTGACAAGCGTTGGGTTTTT
CASP4-qR	ATTAGCTTCACCATGGTGGCTGCCT
IRGM1-qF	TTTAAGAGAAGGAAAACTACTGGAA
IRGM1-qR	GATGACTCGAAGTGCATTGATGAAA
RSAD2-qF	GAGTGGCCTGGCCATCCTGTTCTGC
RSAD2-qR	TGAAGTGGTAGTTGACACTCACGGG
IFIH1-qF	GTGCTGGACCACCTCATCTTTCTGT
IFIH1-qF	CCACGAACATCTGCGTCCATCCCAG
APOBEC3-qF	TCCACTTTAAGAACCTAGGCTATGC
APOBEC3-qR	CTTAAAGACCCCATGGTGAAGGGAG
NAIP2-qF	TTAGTACTGGCTACTGGAAACTGTC
NAIP2-qR	ATCTTCTCTGCTGAGCTCCAGCCTG
NAIP5-qF	CCAGGAGTCTGAGTGAGCAGCTAAG
NAIP5-qR	GCTGGCTGATGTGCTTTGAAATGAT
IRF7-qF	CCCCAGCCGGTGATCTTTCCCAGTC
IRF7-qR	ACTTGCCCATACGCAGGGCCCACAG
β-actin-qF	CGTGACATTAAGGAGAAGCTG
β-actin-qR	CTAGAAGCATTTGCGGTGGAC

### Statistical analysis

All experiments were performed at least three times. Data is presented as mean ± SD. The date of escape latency from MWM were analyzed by multivariate analysis of variance (ANOVA) and the other data were analyzed by one-way ANOVA, followed by Student-Newman-Keuls test. All the analysis was performed by SPSS statistical software (version 21, IBM, Armonk, NY). *P* ≤ 0.05 was considered to be statistically significant (^*^p < 0.05, ^**^p < 0.01, ^***^p < 0.001, ^****^p < 0.0001).

## Results

### HPYD had a better *in Vitro* stability and inhibitory effect on aggregation of Aβ_1−42_ compared to H102

**Figure 1:**
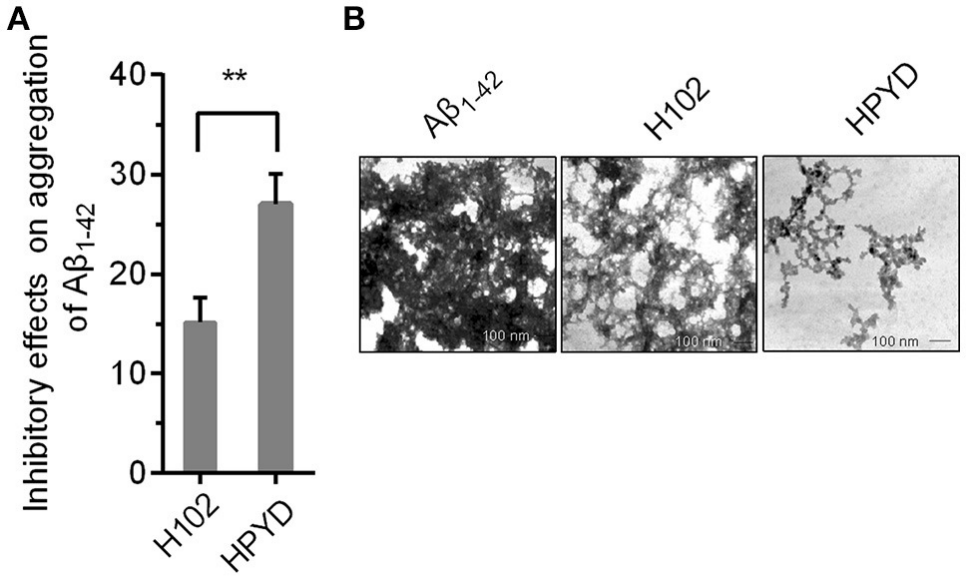
Inhibitory effect of HPYD and H102 on Aβ_1−42_ aggregation. **(A)** ThT fluorescence spectral analysis of the inhibitory effects of HPYD and H102 on Aβ_1−42_ aggregation. **(B)** TEM observations of the inhibitory effects of HPYD and H102 on Aβ_1−42_ aggregation. All of the experiments tests were repeated in triplicates. Statistical significance was denoted with; ^**^p < 0.01.

To investigate the stability of HPYD *in vitro*, accelerated stability test using trypsin was performed. After treatment with 100 mg/L trypsin at 37°C for 400 min, followed by heat treatment for 10 min at 80°C, the concentration of HPYD was 81.67% ± 0.42, while the concentration of H102 was only 41.07% ± 0.17, indicating that the stability of HPYD was better compared to H102 (Table 2). In addition, to compare the inhibitory effects on aggregation of Aβ_1−42_ with H102, ThT fluorescence spectral analysis and TEM was performed. Results showed that HPYD could significantly inhibit the aggregation of Aβ_42_ compared to H102 (Figures 1A,B). Taken together, this demonstrates that HPYD had better *in vitro* stability and inhibitory effects on Aβ_1−42_ aggregation compared to H102.

**Table 2:** The stability of H102 and HPYD by accelerated stability test.

**Variable**	**H102**	**HPYD**
Initial concentration, mg/L	400 ± 0.20	400 ± 1.21
Study time (min)^a^		
0	100 ± 1.28	100 ± 0.55
40	90.01 ± 0.78	96.26 ± 1.13
100	77.92 ± 0.69	91.98 ± 0.36
160	70.62 ± 0.31	91.04 ± 0.62
220	60.46 ± 0.51	91.14 ± 0.83
280	56.26 ± 0.28	85.27 ± 0.57
340	49.60 ± 0.45	81.11 ± 0.96
400	41.07 ± 0.17	81.67 ± 0.42

a *Percentage of initial concentration (Mean ±_SD [%]; n = 3) of H102 (400 mg/L) and HPYD (400 mg/L) remaining after treatment with 100 mg/L trypsin at 37°C for 400 min, followed by heat treatment for 10 min at 80°C*.

### Uptake of FITC-HPYD into the brain after nasal administration

**Figure 2:**
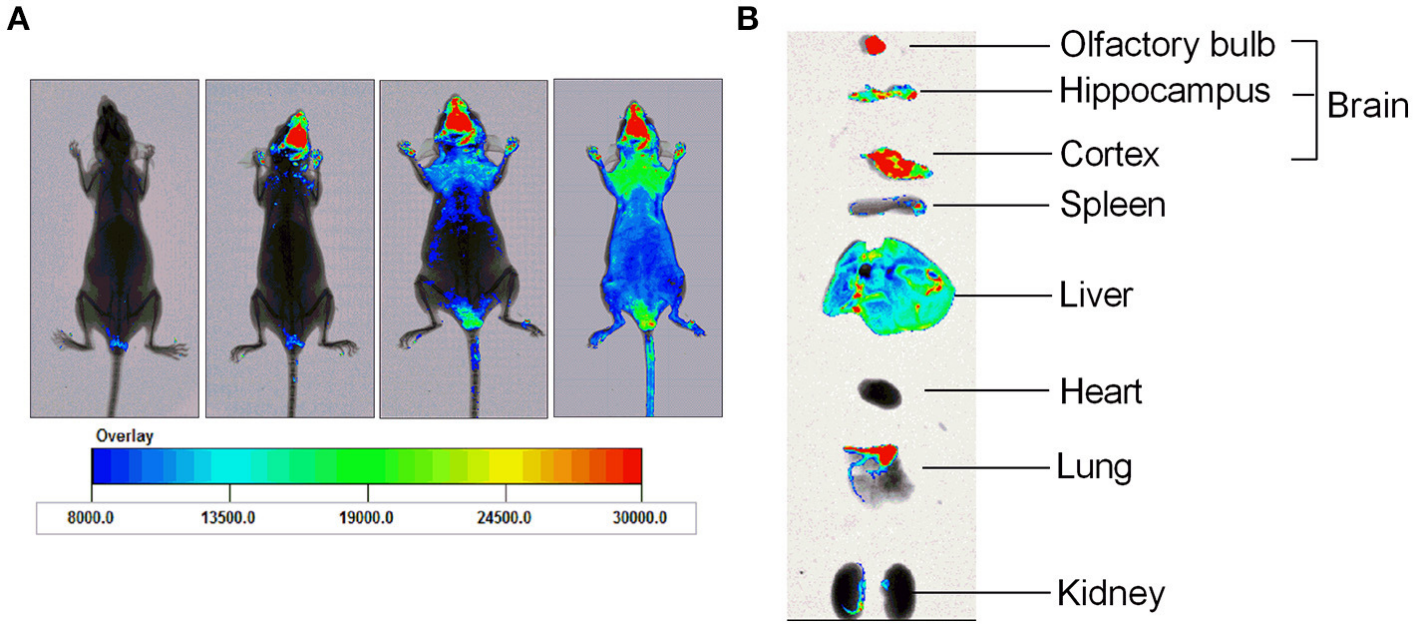
FITC-HPYD transported into brain tissues through nasal administration. **(A)** The *in vivo* distribution of FITC-HPYD at different time points after nasal administration. **(B)** The fluorescence intensity of harvested major organs 30 min after nasal administration.

HPYD, a β-sheet breaker peptide of 10 AAs, is difficult to administer orally or intravenous because it easily undergoes rapid *in vivo* degradation in the gastrointestinal tract and blood. Therefore, to determine whether HPYD can be administered to the brain through the olfactory pathway after nasal administration, HPYD was labeled with FITC and observed by fluorescence imagining of the brain. Before nasal administration, nude mice were irradiated at 490 nm excitation wavelength, and then were subsequently administrated with FITC-HPYD though nasal cavity and irradiated after 5, 15 and 30 min with wavelength of 490 nm. Results showed that FITC-HPYD first appeared at the brain area at 5 min after nasal administration, and the fluorescence gradually expanded with time. As shown in Figure 2A, 30 min after nasal administration, the fluorescence had spread over the whole body of the nude mouse, however, the fluorescence intensity in the brain was the strongest compared to other organs. The mice were then euthanized and the hippocampus, cortex, olfactory bulb, heart, lungs, liver, spleen and kidneys were removed at 30 min after nasal administration and irradiation. The results indicated that the fluorescence intensity of the olfactory bulb was the strongest, with the cortex and the hippocampus having the second and third strongest intensities, respectively. The other major organs showed varying degrees of fluorescence intensity. Among these organs, the lungs had the strongest intensity, followed by the liver (Figure 2B). These results demonstrated that FITC-HPYD could be delivered into the brain through nasal administration.

### HPYD improves the learning and memory ability in APP/PS1 transgenic mice

**Figure 3:**
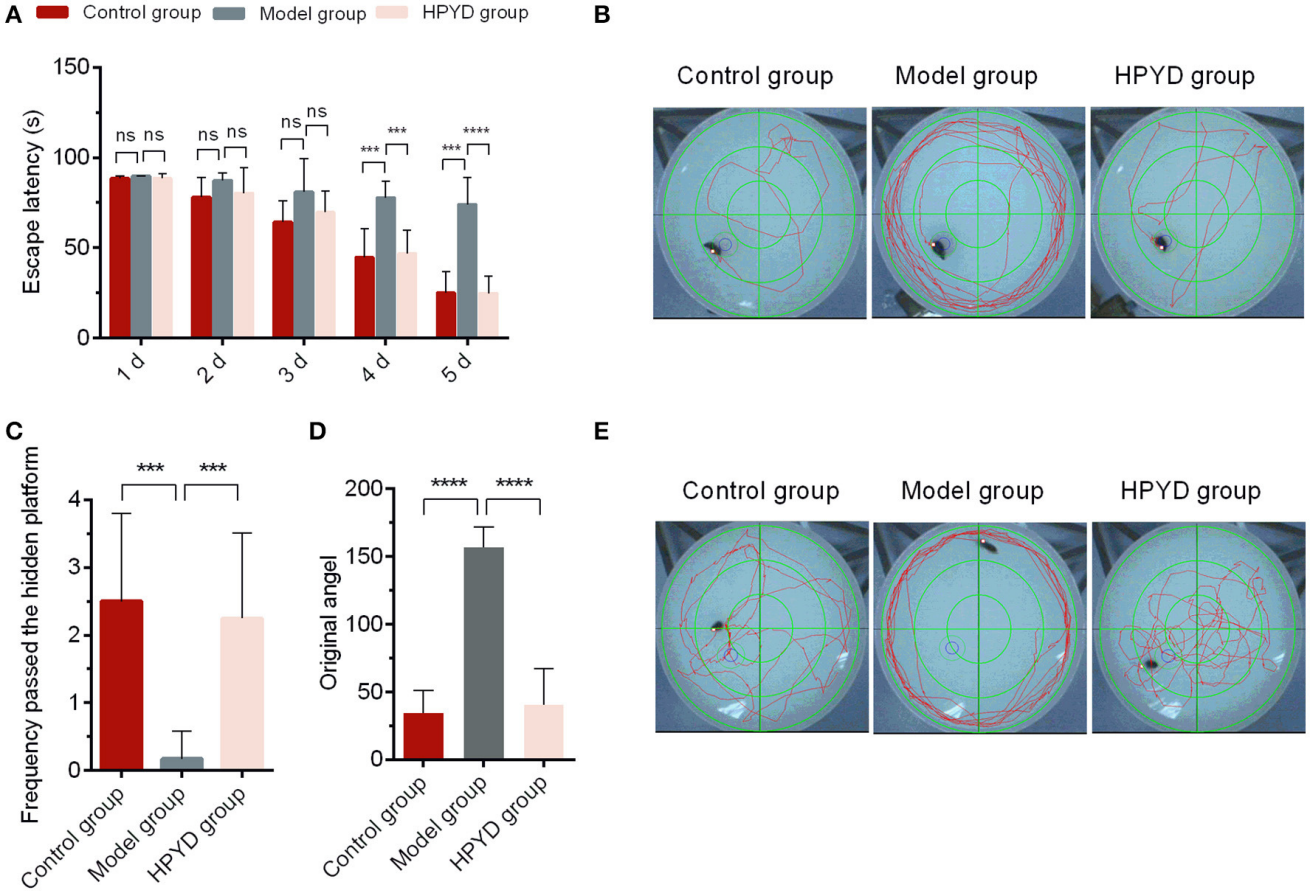
Pathways and behavior of mice in the probe trial. **(A)** Escape latency of mice in the three groups from day 1 to 5. **(B)** The escape-latency of mice obtained through the image capture system of Morris Water Maze test. **(C)** Frequency passed the hidden platform and **(D)** original angle data obtained from the image capture system of the Morris Water Maze test at the Day 6. **(E)** Photographs of spatial probe process obtained from the image capture system of the Morris Water Maze test at Day 6. All of the tests were repeated in triplicates. Statistical significance was denoted with; ^***^p < 0.001; ^****^p < 0.0001; NS, not significant.

MWM test for behavioral analysis is a common strategy to investigate the learning and memory ability in AD mouse models. To determine the efficacy of HPYD on APP/PS1 transgenic mice, the MWM test was conducted for 6 days. First, the escape latency test was performed from Day 1 to Day 5. As shown in Figures 3A,B, the control group and the HPYD group displayed no significant differences from Day 1 to Day 5. In addition, the control group vs. the model group, or the model group vs. the HPYD group, showed no significant difference from Day 1 to the Day 3. However, significant differences were observed between the HPYD group and the model group at Day 4, and in addition, statistical significant differences between the model group and control group were observed on Day 4. The escape latency test indicated that the APP/PS1 transgenic mice, normal mice and the HPYD group mice all required time to find the platform at the start of the test, however, the learning and memory ability demonstrated significant differences after treatment with HPYD in APP/PS1 transgenic mice after 3 days of training. On the 6 day, spatial probe test was performed on the mice without the platform. As shown in Figures 3C–E, memory ability was estimated by the frequency passed the hidden platform (Figure 3C) and original angle (Figure 3D). Frequency passed the hidden platform in the target quadrant displayed significant differences between the model group and the control group, indicating that the model group had a poor ability to find the platform compared to the control group. However, the HPYD group demonstrated an improved ability to find the platform compared to the model group. No significant differences were observed between the HPYD group and the control group. Finally, the ability to locate the original angle was also investigated at the 6th day. The HPYD group was able to find the location of the original angle much quicker compared to the model group, indicating that the HPYD group mice had relatively good memory. No significant differences were observed between the control group and the HPYD group. Collectively, these results indicate that HPYD could improve the learning and memory ability in APP/PS1 transgenic mice after nasal administration of HPYD.

### HPYD reduces Aβ and APP protein levels in APP/PS1 transgenic mice

**Figure 4:**
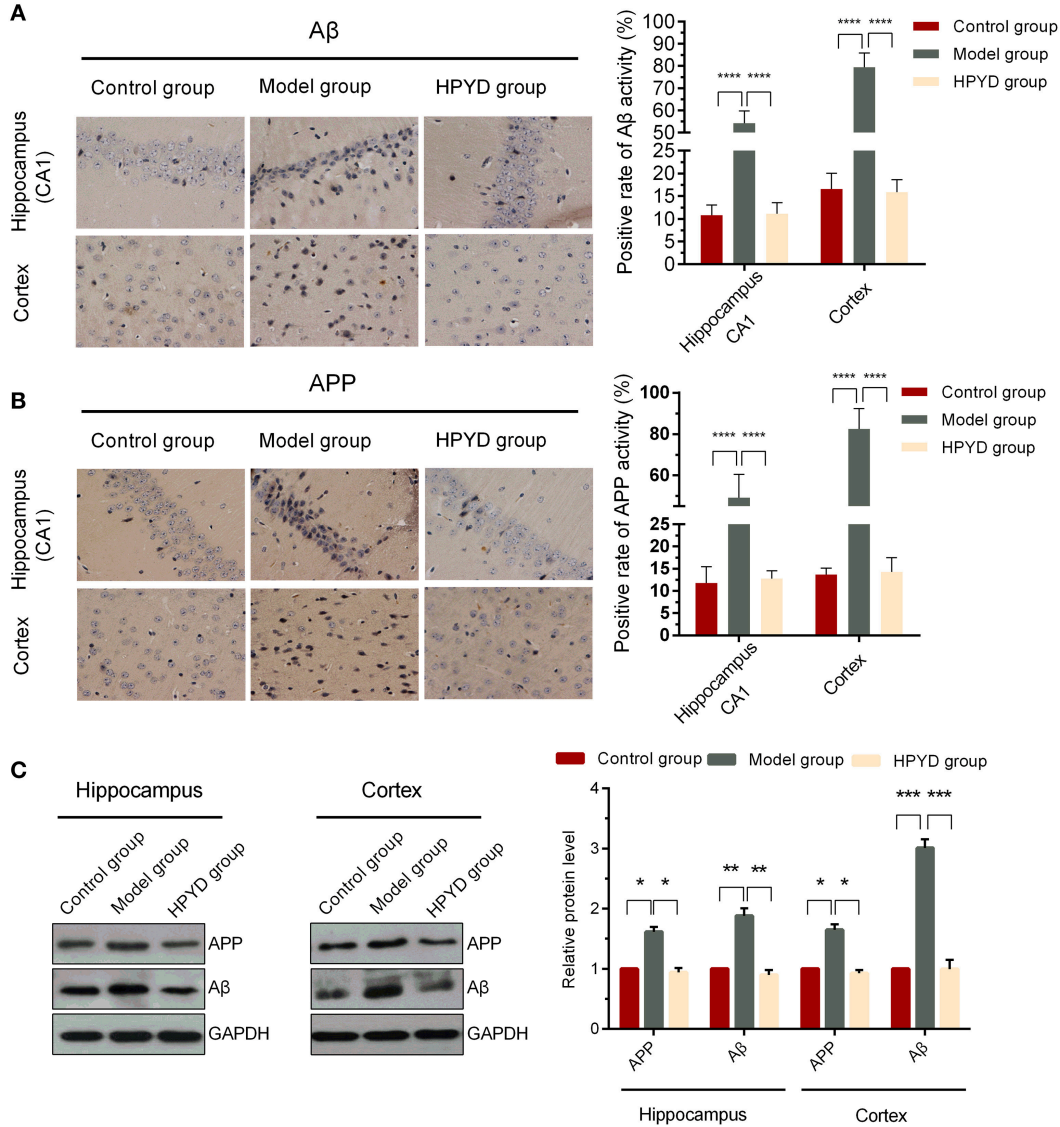
Expression levels of Aβ and APP in the hippocampal CA1 area and cortex. **(A,B)** IHC was performed to determine the expression of Aβ and APP. **(C)** Western blot was used to determine the expression of Aβ and APP. All experiments were repeated in triplicates. Statistical significance was denoted with; ^*^p < 0.05; ^**^p < 0.01; ^***^p < 0.001; ^****^p < 0.0001.

HPYD is a novel β-sheet breaker peptide that could inhibit Aβ_1−42_ aggregation *in vitro*. To determine the effect of HPYD on Aβ *in vivo*, HPYD was administrated though the nasal cavity into the brain. Tissues were then harvested after 6 days for immunohistochemistry and western blot analysis. As shown in Figure 4A, the hippocampus CA1 and cortex had higher expression of Aβ in the model group mice compared to control group mice, while Aβ was lower in the HPYD group compared to the model group. No significant differences were observed between the control group and the HPYD group. In addition, western blot analysis showed that Aβ was highly expressed in the hippocampus and cortex of the model group mice compared with the control group, while Aβ was expressed at a lower level in the HPYD group mice compared to the model group mice (Figure 4B). There were no statistical significant differences between the HPYD group mice and control group mice. APP, the precursor of Aβ, is upregulated in the brain tissues of AD patients. To determine whether HPYD had an effect on APP expression, we determined the expression of APP using immunohistochemistry and western blot (Figures 4B,C). Results showed that APP was upregulated in the brain tissues of the model group mice compared to the control group mice, while it was downregulated in the HPYD group mice compared with the model group mice. The expression of APP in the HPYD group mice was similar to the control group. These results indicate that HPYD can reduce Aβ and APP protein levels in APP/PS1 transgenic mice.

### Transcriptional profiling of brain tissues from APP/PS1 transgenic mice after treatment with HPYD

**Figure 5:**
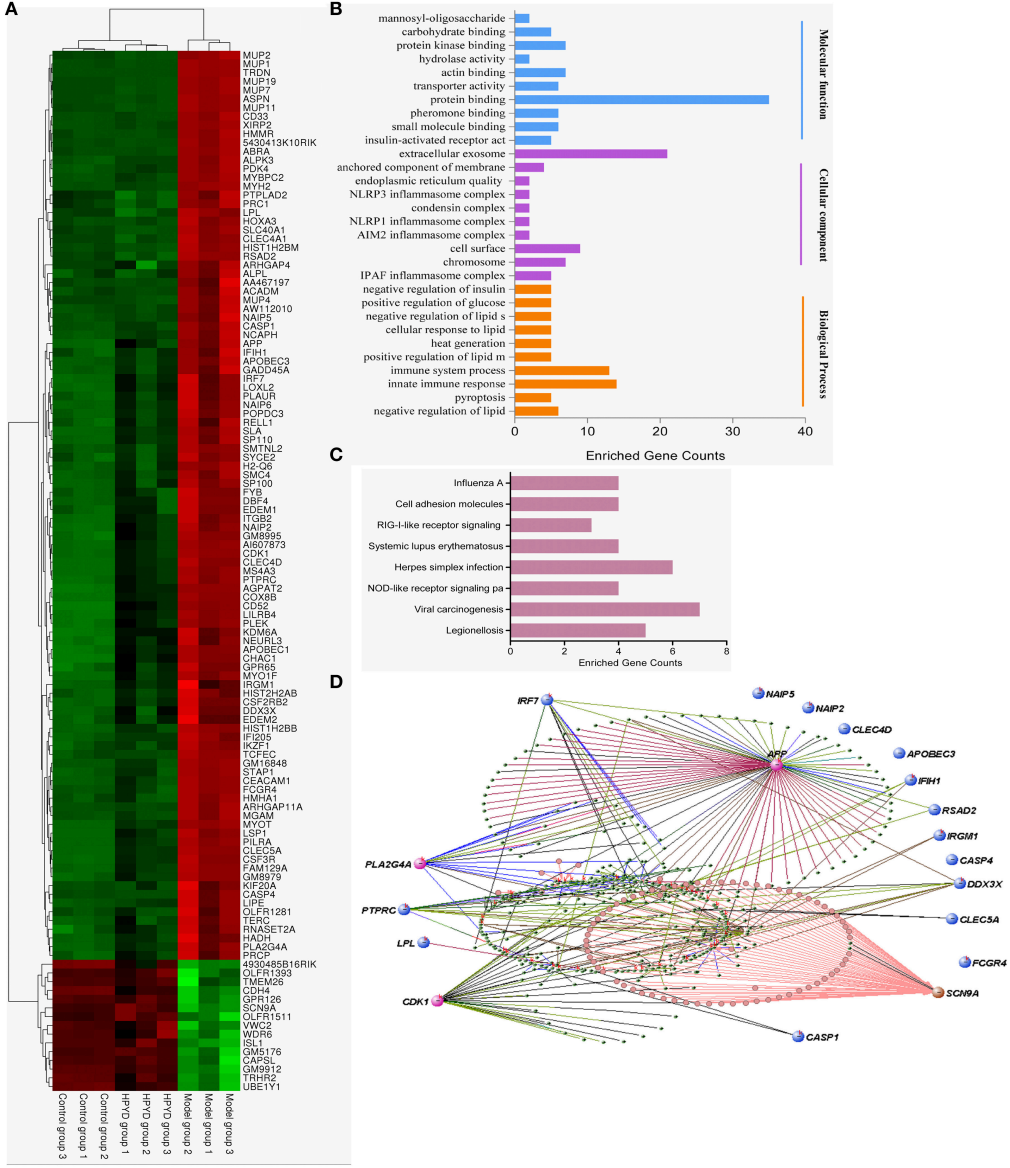
Gene expression profiles after treatment with HPYD. **(A)** Hierarchical clustering of the expression profiles of significantly changed genes after treatment with HPYD. **(B)** The GO terms for molecular functions, cellular components and biological processes were enriched among the significantly changed genes. **(C)** Enriched pathways among the significantly changed genes. **(D)** Co-expression network-based inference of gene-function relationship, node size indicates the node's degree, pink nodes represent genes involve in AD, blue nodes represent genes involve in inflammatory pathway.

**Figure 6:**
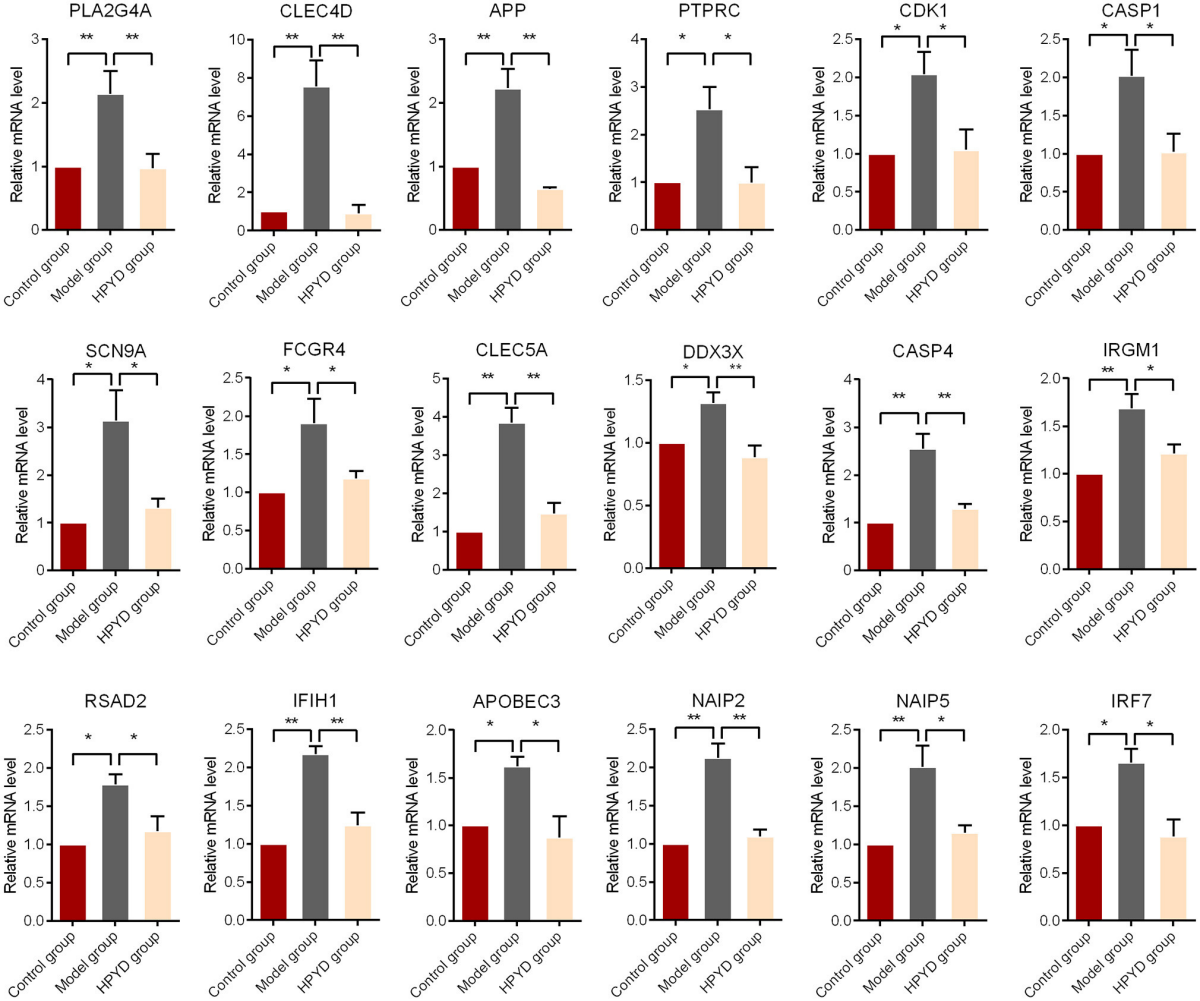
RT-qPCR on the transcriptional levels of 18 selected genes from Figure 5D. All of the experiments tests were repeated in triplicates. Statistical significance was denoted with; ^*^p < 0.05; ^**^p < 0.01.

To investigate the effect of HPYD on gene expression of APP/PS1 transgenic mice, the brain tissues of the control group, model group and HPYD group mice were harvested at the termination of the MWM test. Total RNA from brain tissues were isolated and microarray analysis was performed by Shanghai GMINIX Biotech Limited. Based on microarray analysis, 119 genes with significant differences were selected for further analysis (Figure 5A). Among the selected genes, 15 genes were markedly down-regulated in the model group compared to the control group, however, the expression levels of these genes were restored to normal levels after treatment with HPYD. Additionally, the majority of the selected genes were up-regulated in the model group compared to the control group, which were restored to normal levels after treatment with HPYD. These differentially expressed genes were then analyzed by GO analysis to predict their function. The most enriched GO terms are shown in Figure 5B, and includes; protein binding, extracellular exosome, immune system, inflammatory reaction and gluco-lipid metabolism. KEGG pathway analysis showed that these genes were involved in many inflammatory response pathways, including viral carcinogenesis, RIG-I-like receptor signaling and NOD-like receptor (NLR) signaling pathway (Figures 5C,D). Co-expression network analysis revealed gene-function relationships of the differential gene expressions, including CDK1, PLA2G4A, APP and SCN9A, which were reported to be involved in AD (Ling et al., 2003; Schaeffer et al., 2010; Hilgeroth et al., 2014). In addition, many inflammatory related genes, such as CLEC4D and CLEC5A were differentially expressed. These two genes are members of the C-type lectin/C-type lectin-like domain (CTL/CTLD) superfamily that play important roles in inflammation and immune response (Wu et al., 2013; Wilson et al., 2015). Other genes of interest include; Inflammasome-related CASP1 (encodes caspase-1) and CASP4 (encodes caspase-4) that mediates non-canonical activation of the NLRP3 inflammasome (Schmid-Burgk et al., 2015). NAIP2 and NAIP5, belong to the NAIP/NLRC4 inflammasomes, and play important physiological roles in antibacterial defense and inflammation (Diebolder et al., 2015; Zhang et al., 2015). In addition, 18 genes associated with AD or inflammatory responses were selected for validation by RT-qPCR. There was a good concordance in the expression levels of these genes by RT-qPCR and microarray (Figure 6). Taken together, the above results indicated that dysregulated expression of several genes in the model group mice could be restored to normal levels after treatment with HPYD, indicating that HPYD may be a potential therapeutic candidate for treatment of AD.

## Discussion

Alzheimer's disease is the most common dementing disease worldwide. Over the past decade, several potential therapeutics were evaluated in clinical trials, but only five drugs (Memantine and four cholinesterase inhibitors) have been approved worldwide to treat AD (Cummings et al., 2016). However, due to the modest clinical efficacy on temporarily ameliorating memory and thought, these five drugs are not very efficacious in treating the underlying cause of AD and preventing the rate of cognitive decline. Therefore, it is critical to develop novel drugs for the treatment of AD.

The amyloid hypothesis is one of the most accepted mechanistic hypothesis for AD posits that dysregulated aggregation and accumulation of Aβ in the brain leads to nerve fiber entanglement and neuronal apoptosis (Hardy and Selkoe, 2002). The amyloid hypothesis implies that decreasing the levels of Aβ or inhibiting Aβ aggregation could lead to a cure for AD. Based on this hypothesis, many drugs were developed for the treatment of AD. Tramiprosate, a drug of potential interest for the treatment of AD, inhibits Aβ aggregation and amyloid deposition (Gervais et al., 2007). Bapineuzumab and solanezumab, two humanized monoclonal antibodies that specifically target amino acids 1–5 or 16–24 of Aβ peptide, increases the clearance of Aβ and decreases amyloid deposition in the brain (Salloway et al., 2014). Despite efficacy in AD animal models, the above mentioned drugs all failed in Phase 3 clinical trials due to efficacy. However, the failure of several amyloidocentric drugs for the treatment of AD did not diminish the pathogenic theory of this disease. Numerous novel drugs have been designed or screened for inhibiting Aβ production. For example, Icariside II (ICS II), an anti-cancer natural compound extracted from *Herba Epimedii* Maxim, was demonstrated to inhibit Aβ production by reducing APP and BACE1 expression in APP/PS1 transgenic mice (Yan et al., 2017). ALZ-801, a novel prodrug of tramiprosate, has been shown to have excellent oral safety and tolerability compared to tramiprosate though phase I studies (Hey et al., 2017).

Previously, we designed a β-sheet breaker peptide, H102, that could inhibit Aβ aggregation as well as reducing amyloid load in the brains of APP/PS1 transgenic mice, thus limiting brain damage and improving the symptoms of AD in animal models (He et al., 2008; Lin et al., 2014). However, H102 was not stable *in vitro*. We designed alternative β-sheet breaker peptide, HPYD, by substituting Phe7 with Tyr7. This peptide showed higher *in vitro* stability and better efficacy of inhibiting Aβ aggregation compared to H102. In addition, FITC-labeled HPYD demonstrated that the peptide could enter the brain after nasal administration. MWM test demonstrated significant spatial learning and memory disorders in APP/PS1 transgenic model mice, which were in concordance with previous studies (Yan et al., 2017). Several studies have also demonstrated that Aβ and APP protein expression levels were markedly increased in APP/PS1 transgenic model mice. These outcomes were effectively reversed by treatment with HPYD through nasal administration. Our results indicate that HPYD may be a potential therapeutic candidate for the treatment of AD.

To further demonstrate that HPYD is a potential therapeutic for AD, we analyzed the mRNA levels of APP/PS1 transgenic mice to investigate the effect of HPYD on the brain. APP/PS1 transgenic model mice have early memory dysfunctions even before the degeneration of synapses and neurons (Dickey et al., 2003). The mRNA levels of many genes in APP/PS1 transgenic model mice are dysregulated, but are restored to normal levels after treatment with HPYD. The 119 genes that were differentially expressed were involved in inflammatory reaction, gluco-lipid metabolism and other pathways. Neuro-inflammation has been recognized as playing an important role in the pathogenesis of AD (Cacquevel et al., 2004; Sagy-Bross et al., 2013), which is mediated by microglia (MG) that can participate in the immune response, leading to increase in pro-inflammatory cytokines and chemokines (Lucin and Wyss-Coray, 2009; Prinz et al., 2011). Inflammasomes are responsible for the maturation and release of pro-inflammatory cytokines and the activation of an inflammatory form of cell death. NLRP3 inflammasome, one of the most widely studied members of the NLR family, can be activated by Aβ and enhances AD progression by mediating a detrimental chronic inflammatory tissue response (Heneka et al., 2013). Our results demonstrated that inflammasome-related CASP1 and CASP4, which mediates non-canonical activation of the NLRP3 inflammasome, was highly expressed in APP/PS1 transgenic model mice, which was in concordance with previous studies (Schmid-Burgk et al., 2015). Inflammatory factors, like APOBEC3, CLEC4D, DDX3X, FCGR4, IFIH1, IRF7, NAIP2, NAIP5, CLEC5A, PTPRC and PLA2G4A, were also overexpressed in APP/PS1 transgenic model mice. PLA2G4A has been demonstrated to mediate apoptotic neuronal death in AD brain and could be induced by aggregated Aβ peptide_1−42_ (Sagy-Bross et al., 2013). PTPRC (CD45) was upregulated in microglial and has been association with AD (Masliah et al., 1991). In addition, we also observed that cell-cycle protein CDK1 (CDC2) was upregulated in APP/PS1 transgenic model mice, which is associated with the pathogenesis of AD (Johansson et al., 2005). Most importantly, our study also found that the disordered changes in neurons were effectively restored by treatment with HPYD through nasal administration, indicating HPYD as a potential therapeutic for AD.

In summary, we designed a new β-sheet breaker peptide HPYD, which showed better *in vitro* stability and inhibitory effects on Aβ_1−42_ aggregation compared to H102. HPYD could improve the learning and memory ability in APP/PS1 transgenic mice by reducing Aβ and APP protein levels. Microarray analyses demonstrated that dysregulated gene expression in model mice could be restored to normal levels after treatment with HPYD by inhibiting the aggregation of Aβ. This provides a novel therapeutic strategy for the treatment of AD.

## Author contributions

SX conceived project, WL, FS, and SX designed the study and wrote the paper. WL and FS performed the experiments. MW, FJ, XB, and LL provided technical assistance and contributed to the preparation of the figures and manuscript. HT contributed to English editing and academic writing in the whole manuscript. All authors reviewed the results and approved the final version of the manuscript.

### Conflict of interest statement

The authors declare that the research was conducted in the absence of any commercial or financial relationships that could be construed as a potential conflict of interest.
